# A Decision Support Tool Facilitating Medicine Design for Optimal Acceptability in The Older Population

**DOI:** 10.1007/s11095-018-2424-3

**Published:** 2018-05-07

**Authors:** Thibault Vallet, Emilie Belissa, Sandra Laribe-Caget, Alain Chevallier, François-Xavier Chedhomme, Patrick Leglise, Matthieu Piccoli, Hugues Michelon, Vanessa Bloch, Sylvie Meaume, Anne-Sophie Grancher, Nathalie Bachalat, Imad Boulaich, Fattima Abdallah, Maite Rabus, Jean-Paul Rwabihama, Annie-Claude Ribemont, Celia Lachuer, Ines Perquy, Laurent Lechowski, Anne Delahaye, Mathieu Depoisson, Yann Orven, Caroline Guinot, Stephane Gibaud, Corinne Michel, Abdel Mahiou, Sid-Ahmed Belbachir, Jean-Hugues Trouvin, Amelie Dufaÿ-Wojcicki, Vincent Boudy, Fabrice Ruiz

**Affiliations:** 1 ClinSearch, 110 Avenue Pierre Brossolette, 92240 Malakoff, France; 2 Département Recherche et Développement Pharmaceutique, Agence Générale des Equipements et Produits de Santé (AGEPS), Assistance Publique-Hôpitaux de Paris (AP-HP), Paris, France; 3 Hôpital Rothschild, Groupe Hospitalier Universitaire Est Parisien, Assistance Publique-Hôpitaux de Paris (AP-HP), Paris, France; 4 Hôpital Broca, Groupe Hospitalier Universitaire Paris Centre, Assistance Publique-Hôpitaux de Paris (APHP), Paris, France; 5 Hôpital Joffre Dupuytren, Groupe Hospitalier Universitaire Henri Mondor, Assistance Publique-Hôpitaux de Paris (AP-HP), Draveil, France; 6 Hôpital Sainte Périne, Groupe Hospitalier Universitaire Paris Ile-de-France Ouest, Assistance Publique- Hôpitaux de Paris (AP-HP), Paris, France; 7 Hôpital Fernand Widal, Groupe Hospitalier Universitaire Saint-Louis – Lariboisière – Fernand-Widal, Assistance Publique-Hôpitaux de Paris (AP-HP), Paris, France; 8 Université Paris Descartes, Paris, France; 9 Hôpital Vaugirard, Groupe Hospitalier Universitaire Paris Ouest, Assistance Publique-Hôpitaux de Paris (AP-HP), Paris, France; 10 Centre Hospitalier de l’Ouest Vosgien, Neufchâteau, France; 11 Hôpital René Muret, Groupe Hospitalier Universitaire Paris Seine-Saint-Denis, Assistance Publique-Hôpitaux de Paris (AP-HP), Sevran, France

**Keywords:** drug formulation, elderly, medicine acceptability, multivariate analysis, swallowability

## Abstract

**Purpose:**

Medicine acceptability, which is of the utmost importance for vulnerable patients’ adherence, is driven by both user and product characteristics. Herein, a novel multivariate approach integrating the many aspects of acceptability is used to discriminate positively and negatively accepted medicines in the older population.

**Methods:**

An observational study was carried out in eight hospitals and eight nursing homes to collect a large set of real-life data on medicines uses in older patients (≥65 years). Mapping and clustering explored these multiple observational measures and summarised the main information into an intelligible reference framework. Resampling statistics were used to validate the model’s reliability.

**Results:**

A three-dimensional map and two clusters defining acceptability profiles, as positive or negative, emerged from the 1079 evaluations. Factors of interest (medicines, user features…) were positioned on the map at the barycentre of their evaluations and assigned to an acceptability profile. Focusing on patients’ ability to swallow, we have highlighted the tool’s efficacy in demonstrating the impact of user features on medicine acceptability.

**Conclusions:**

This multivariate approach provides a relevant judgement criterion for this multi-dimensional concept. Facilitating the choice of the most appropriate dosage form to achieve optimal acceptability in a targeted population, this tool is of real potential to improve clinical decisions.

## Introduction

Physical and cognitive deterioration due to aging may negatively impact the safety and efficacy of some medications. Swallowing disorders are one such age-related alteration affecting solid oral dosage form (SODF) administration (1–3). Crushing tablets and opening capsules are commonly used to achieve administration, despite pharmacokinetic/pharmacodynamic issues that may induce dosage errors (4–12).

Recently, regulatory authorities such as the European Medicine Agency (EMA) (13,14), the Food and Drug Administration (FDA) (15) and the International Council for Harmonization (ICH) (16) raised the importance to develop medicinal products well suited to the characteristics of the targeted patients, especially in frail populations. In this context medicines acceptability has been identified as a key factor of the patient adherence. Acceptability could be defined as “an overall ability of the patient and caregiver (defined as ‘user’) to use a medicinal product as intended (or authorised)” (17). The EMA defined acceptability as a multi-dimensional concept driven by characteristics of both medicines and users, be they paediatric and older populations (13,14).

Various methods have been used to assess medicine acceptability in clinical studies (18), however none of the referenced studies simultaneously considered the many different dimensions of acceptability (swallowability, palatability, complexity of use,…). To bridge this gap a multivariate approach has been developed and tested in the paediatric population (19,20). Based on a large set of medicine use evaluations combining multiple observational measures, an acceptability reference framework allows discrimination between positively and negatively accepted medicines in distinct subpopulations of patients.

This paper presents the development of an acceptability reference framework in the older population using this methodology. The study aimed to confirm the validity of this multivariate approach and to develop a decision support tool providing a judgment criterion for a multi-dimensional concept.

## Materials and Methods

### Study Design

A multicentre, prospective, cross-sectional, and strictly observational study was conducted in France between October 2016 and November 2017. This study was carried out in collaboration with a network of physicians and pharmacists in eight hospitals and eight nursing homes. The study focused on any medicine use in older patients (65 years and over), with the exception of the infusions with a catheter already present, considering that the insertion of the catheter belongs to the administration sequence of the medicine. Patients were recruited at random on a voluntary basis in the recruiting centres. Approvals were obtained from the French advisory committee for data processing in health research and the data protection authority.

The multivariate approach aimed to design a model that fits with real-life data reflecting different users’ behaviours for various medicines. A massive data set would be needed to thoroughly encompass this multi-dimensional concept due to the large variety of users and the wide range of products on the market. However paediatric results have demonstrated the reference framework reliability with 680 evaluations (20).

### Data Collection

The healthcare professional observing the first medicine use following study inclusion (oral participation agreement) filled in a standardised web-questionnaire, which consists of measures describing acceptability and information explaining acceptability.

#### Measures Describing Acceptability

Some patients from paediatric and older populations are unable to provide reliable and valid self-evaluations due to development or deterioration of physical and cognitive abilities. To standardise data collection in these populations we used observer reports that include only those events or behaviors that can be observed as encouraged (21,22). Observers had to report the result of the intake (fully, partly or not taken); the patient’s reaction (positive, neutral or negative reaction); the time needed to prepare - starting from the opening of the cardboard box - and to administrate - starting from the moment it is ready to use - the prescribed dose of medication. This discrete variable (10-s accuracy) was transformed into a categorical one (Short, Medium or Long time), based on data distribution and clinical practice expertise of the authors. Furthermore, the use of any of the following methods to achieve administration was recorded resulting in four dichotomous variables (use or non-use): alteration of the intended use (manipulate dosage form such as tablet crushed or capsule opened; use a device not provided; use another route/mode of administration); divide the intake of the required dose; use drink, texture-modified water or food to mask the taste or ease swallowing; use of restraint. Each evaluation of one medicine taken by one patient corresponded to a particular combination of observed measures (categories) for the seven observational variables. These variables were included in the analysis without weighting in order to describe the overall ability and willingness of patients and caregivers to use (prepare and administer) any medicine as intended.

#### Factors Affecting Acceptability

Each evaluation was related to many explanatory variables in order to investigate their impact on acceptability and consequently, to highlight factors affecting acceptability.

Observers reported the following information on medicine use circumstances: the person(s) in charge of preparing and administrating the medicine (patient, healthcare professionals, and/or other caregiver), the location, and time of day. They were also required to report the exact name of the medicine under investigation (Brand name + Strength + Pharmaceutical form), selected from a list specifying all medicinal products available on the French market, and some information on the treatment such as the required dose, the required dosing frequency, the treatment duration, the disease/symptom treated, and the number of concomitant medications. Thereafter, additional data on the medicine under investigation were extracted from the summary of product characteristics (SmPC) such as the active pharmaceutical ingredient (API), the classification of API using the Anatomical Therapeutic Chemical classification system (ATC), the excipients (e.g. flavour and sweetener), the physical characteristics (e.g. size and colour), the method of administration, the nature of device (if any) or the nature and contents of container.

Information on the patient were also collected: socio-demographic characteristics (gender, age and weight), living situation, treatment history (first, occasional, or ongoing treatment with the medicine), comorbidities (coded using MedDRA, a standardised international medical terminology, as well as diseases), and disabilities (swallowing disorders, muscular or rheumatologic disorders of the upper limbs, or memory disorders). Furthermore, the Lawton’s Instrumental Activities of Daily Living (IADL) scale (23) with 4 items (ability to use telephone, mode of transportation, responsibility for own medications, and ability to handle finances) was used to evaluate the patient’s autonomy. For a specific research issue, the Fried frailty phenotype (24) and the Mini-Mental State Examination (MMSE) (25) were collected in a specific recruiting centre.

Regarding the informant, we collected the occupation of the healthcare professional (doctor, intern, non-resident, pharmacist, nurse, research associate or other).

To illustrate explorations of factors affecting acceptability, we focused on the influence of swallowing disorders on acceptability of a particular medicine anonymously labelled “Y”.

### Data Analysis

#### Acceptability Reference Framework

To develop the acceptability reference framework we followed the multivariate data analysis procedure previously published (19,20) as described briefly hereafter.

A Multiple Correspondence Analysis (MCA) was used for the mapping process. This factorial method was performed on a data table where each row corresponded to one medicine taken by one patient (e.g. patient n°10 taking the medicine “Y”), each column represented one of the seven observational variables (e.g. result of the intake), with an observed measure (e.g. fully taken) being entered into each corresponding cell. The key relationships between the observed measures were summarised and visualised by the MCA that provided an acceptability map in an intelligible, low-dimensional space. The dimensions of the map revealed those associations and dissociations of observed measures that most contributed to explaining variability observed in the data. Thus, the map highlighted the major information in terms of medicine acceptability variation.

Subsequently, hierarchical clustering on principal components and k-means consolidation gathered the evaluations into clusters defining distinct acceptability profiles. The clusters were described by the categories significantly over-represented into their subset of evaluations in comparison to a random distribution: v-test value greater than 1.96 (*p*-value<0.05). The higher the v-test value, the more strongly the category was over-represented in the cluster.

The R packages “FactoMineR” (26) and “MissMDA” (27) were used to perform multivariate analysis and to handle missing data, respectively.

#### Model Reliability

To validate the statistical reliability of the acceptability reference framework we used resampling statistics (20).

To demonstrate the significance of the percentage of data set variance summarised by the map (inertia) we used a non-parametric statistical significance test: permutation testing (28). Inertia distribution under the null hypothesis - rearrangement of labels among the observed data has no effect on the test statistic - was estimated using 10,000 rearrangements of data. For each round, rearrangement of the categories was performed at random for the seven constituting variables independently, then mapping was performed and the inertia explained by the newly created map was recorded. The null hypothesis could be disproved, if the proportion of rearrangement with an inertia value at least as extreme as the observed statistic was less than the 5% significance level.

To explore the impact on the model of variations in the data we used bootstrapping (29). Ten thousand rounds of resampling were performed. For each simulated data set a new reference framework was created, then statistical indicators measured change compared with the original one based on observed data. The RV coefficient (30) measured the closeness between the categories’ coordinates on the maps, and the Jaccard coefficient (31) measured the similarity between the subsets of evaluations gathered into the clusters. For each original cluster, the maximum Jaccard coefficients indicated the most similar cluster found among the new ones. The values ranged from 0 to 1 for both indicators. We used FactoMineR to demonstrate the significance of the RV coefficient (32), while a Jaccard coefficient value superior or equal to 0.75 denotes a ‘good recovery’ of the cluster. We averaged the values over all the resampling rounds and computed the 95% confidence interval around the mean to quantify the dispersion.

#### Acceptability Scoring

To assess medicine acceptability using the reference framework we followed the procedure previously published (19,20) as described hereafter.

The barycentre of the evaluations related to a particular medicine defined its position on the map. The medicine was assigned to the cluster with the nearest barycentre, which defined its acceptability profile. The profiles zones were plotted on the acceptability map using simulations of all the possible barycentre positions. Confidence ellipses surrounding the barycentre for all dimension pairs defined an area containing its true position with 90% probability if the experiment were to be repeated. Each ellipse was made of 1000 points. Each point was assigned to one of the clusters. The proportion of points belonging to the different clusters were then recorded. The acceptability score was structured by the acceptability profile of the barycentre and the proportion of confidence ellipses belonging to it. We consider that a minimum of 30 evaluations are required to obtain a reliable score. Acceptability scores were significantly different if confidence ellipses did not overlap on the map.

Similarly, acceptability scoring may be performed for any product and user characteristic in order to explore factors affecting acceptability.

## Results

### Study Participation

There were 1079 patients included in the study. Table I presents the demographic characteristics of these patients from 65 years of age to centenarians.

**Table I:** Demographic Characteristics of the Patients

Characteristics of patients (*n* = 1079)		n	(%)
Gender	Women	758	(71)
	Men	307	(29)
	*md* ^*a*^ *: 14*		
Age (years) *Mean: 86.4 sd(7.2)*	[65, 75)	67	(6)
	[75, 85)	324	(30)
	[85, 95)	556	(52)
	[95, 104]	126	(12)
	*md: 6*		
Place	Hospital	848	(79)
	Nursing home	231	(21)
Disabilities	Swallowing disorder	187	(18)
	Muscular or rheumatologic disorders of the upper limbs	261	(25)
	Memory disorder	614	(58)
IADL Scale^b^	4	126	(12)
	3	119	(11)
	2	153	(14)
	1	323	(31)
	0	320	(32)
	*md: 38*		
Number of prescribed medicines	[0, 5)	81	(8)
	[5, 10)	428	(41)
	[10, +∞)	537	(51)
	*md: 33*		

^a^md: missing data

^b^Overall score calculated as a sum of the four items. For each item, a score of 1 defined an autonomous patient while a score of 0 a dependent one

Table II presents the characteristics of the 280 distinct medicinal products assessed: 59% were assessed only once, while 3% were assessed 30 times or more. Considering, the fifth level of the ATC code, there were 125 distinct API in the sample of medicines.

**Table II:** Characteristics of the Medicines

Characteristics of medicines (*n* = 280)		n	(%)
Formulations	Divisible tablet	39	(14)
	Coated tablet	38	(14)
	Divisible coated tablet	36	(13)
	Capsule	35	(13)
	Tablet	20	(7)
	Orally disintegrating tablet	14	(5)
	*Other (2% < n < 5%): oral solution, powder for oral solution, capsule sustained release, oral solution in drops, solution for injection.*		
	*Other (n ≤ 2%): coated tablet sustained release, tablet sustained release, effervescent tablet, dispersible tablet, oral suspension, powder for oral suspension, patch, enrobed tablet, collyre, syrup, capsule gastro-resistant, tablet gastro-resistant, suspension for inhalation, granule sustained release, oral gel, divisible coated tablet sustained release, gastro-resistant enrobed tablet, lozenge.*		
Anatomic therapeutic subgroups (ATC2)	Analgesics (N02)	41	(15)
	Psycholeptics (N05)	39	(14)
	Psychoanaleptics (N06)	32	(12)
	Antiepileptics (N03)	23	(8)
	Drugs used in diabetes (A10)	21	(8)
	Antithrombotic agents (B01)	10	(5)
	*Other (2% < n < 5%): Agents acting on the renin-angiotensin system (C09), Beta blocking agents (C07), Anti-parkinson drugs (N04), Drugs for constipation (A06), Mineral supplements (A12), Calcium channel blockers (C08), Drugs for acid related disorders (A02), Thyroid therapy (H03).*		
	*Other (n ≤ 2%): Antianemic preparations (B03), Ophthalmologicals (S01), Antibacterials for systemic use (J01), Lipid modifying agents (C10), Antihypertensives (C02), Urologicals (G04), Cardiac therapy (C01), Diuretics (C03), Other nervous system drugs (N07), Antivirals for systemic use (J05), Vasoprotectives (C05), Endocrine therapy (L02), Muscle relaxants (M03), Drugs for functional gastrointestinal disorders (A03), Drugs for obstructive airway diseases (R03), Corticosteroids for systemic use (H02), Antimycobacterials (J04), Anti-inflammatory and antirheumatic products (M01), Antihemorrhagics (B02).*		

### Measures Describing Acceptability

The 1079 evaluations were comprised of 106 distinct combinations of categories, of the 432 mathematically possibilities, and reflected existing users’ behaviours. The following ideal combination reflecting a medicine use without any problem was the most used (32% of the evaluations): “Fully taken”, “Neutral reaction”, “Short time”, “No divided dose”, “No alteration”, “No food drink” and “No restraint”. The same combination with the category “Medium time” and “Long time” to replace “Short time” were the second (14%) and the third (8%) most observed, respectively. The median preparation and administration time was 30 s. There were 6 missing data (md). The preparation and administration time was transformed into a categorical variable with three categories corresponding to “Short time” (20 s and less - 44% of the evaluations), “Medium time” (from 30 s to 1 min - 34%) and “Long time” (more than 1 min - 22%).

The following combination of the worst categories was used only once: “Not taken”, “Negative reaction”, “Long time”, “Use divided dose”, “Use alteration”, “Use food drink” and “Use restraint”. These negatively connotated categories were used less frequently than the others. Indeed, none of the prescribed dose was taken in only 0.4% of evaluations and a partial dose was taken for 3.5%, while the required dose was fully taken in 96.1% of cases (md: 27). Regarding the patient’s reaction, 76% of the evaluations were neutral while 11% positive and 13% negative (md: 6).

The results from the methods used to achieve administration revealed that the required dose was divided for 13% of the patients as it could not be taken whole (md: 1), in 19% of the evaluations food or drink were used, either mixed with drug or taken just before or after administration, to mask the taste or ease swallowing (md: 1), and 5% of the patients forced themselves to take the medicine (md: 1). For 10% of the evaluations a form modification, predominantly crushing tablets or opening capsules, was performed before administration. For 3% a device not provided with the medication was used to perform administration (those using a similar device in institution for hygienic reasons were not counted). For 3% an unintended route/mode of administration was used, mainly oral administration of an injectable solution, swallowable tablet chewed, and orally disintegrating tablet swallowed. These alterations of the use were merged in a binary variable employed in 14% of the evaluations (md: 1). There were only 0.6% of missing data.

### Acceptability Reference Framework

**Fig. 1:**
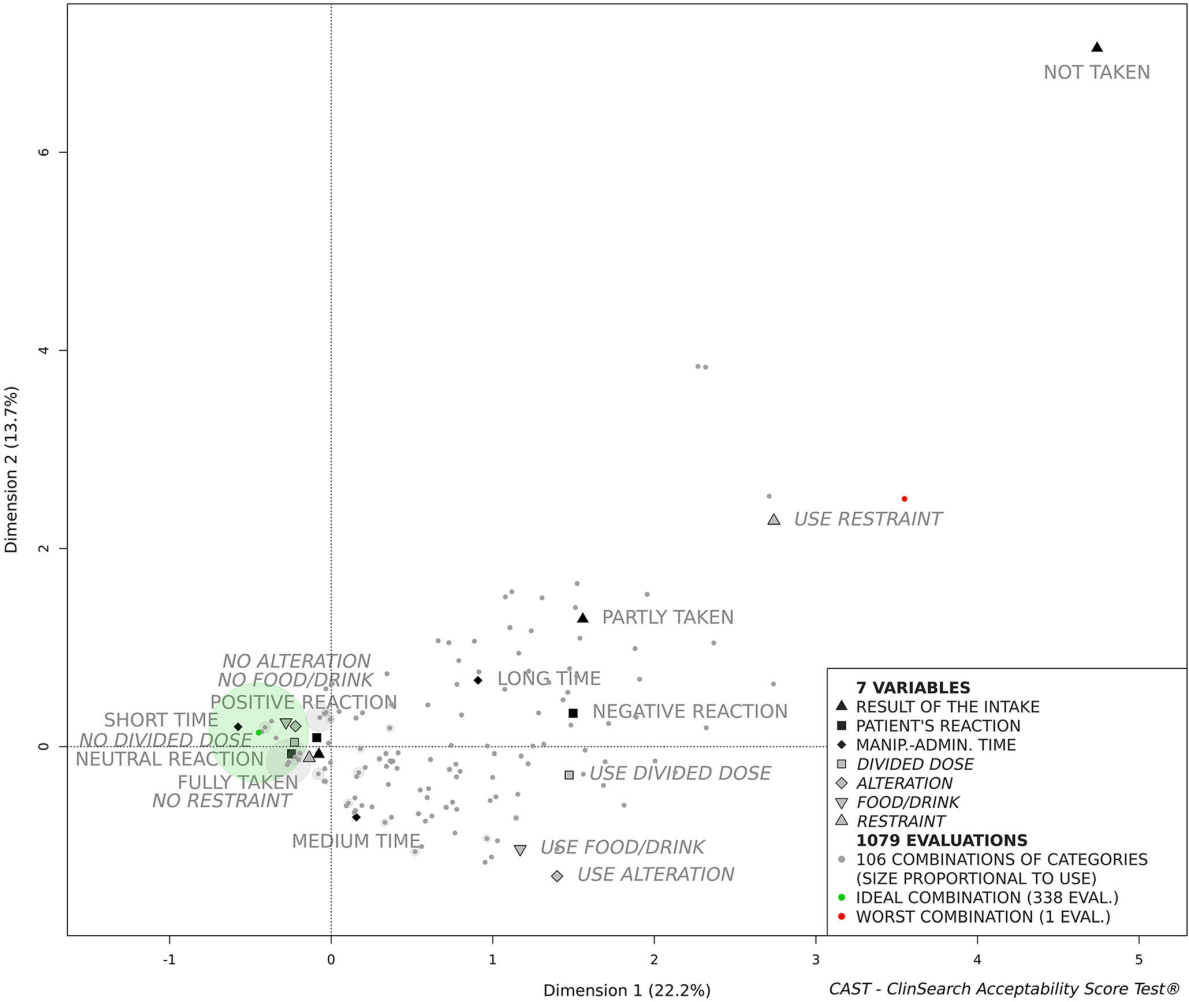
Acceptability map

The MCA explained the total variance of the data set with 10 dimensions. The first dimensions extracted the observed measures associations that contribute the most in explaining the variations in the data set while the last ones are restricted to noise. The acceptability map is comprised of the first three-dimensions, as they accounted for 47.2% of the data set variance in a stable and intelligible form. Figure 1 presents the first two dimensions of the acceptability map, although it remains a three-dimensional space for the following clustering and scoring processes.

The zero of the map is the barycentre of all categories and evaluations. The less often the category appeared as a response, the furthest it was placed from the zero of the map. Thus, the category “Not taken” selected for only 0.4% of the evaluations is the furthest point from zero. Proximity between categories revealed that they were often selected together in the evaluations. For example, the categories “Fully taken” and “No restraint” were often selected together, but rarely with “Long time” and “Negative reaction”. Similarly, evaluations completed in a comparable manner, converged on the map. Figure 1 illustrates how the ideal and the worst combinations were positioned onto the map. Between these extreme combinations the remaining 104 combinations were positioned according to their similarity.

The dimensions structure the acceptability map. The first dimension that juxtaposes the positively connoted categories on the left side of the map (dimension 1 negative coordinates) to the categories that are negatively connoted on the right side (dimension 1 positive coordinates). On the right side of the map (Fig. 1) the second dimension opposes those evaluations related to “Use alteration”, “Use food/drink” and “Medium time” placed at negative coordinates at the bottom of the map to those related to the negatively connoted categories “Use restraint”, “Not taken”, “Long time” and “Partly taken” on the top (dimension 2 positive coordinates). The third dimension illustrates the contrast (on the left side of the map, Fig. 1) between those evaluations related to “Positive reaction” and “Medium time” (dimension 3 negative coordinates) to those related to “Short time” and “Neutral reaction” (dimension 3 positive coordinates).

The set of evaluations was partitioned into two meaningful clusters characterised by the categories significantly over-represented into each of them (Table III). All the categories positively connoted were over-represented in the first cluster defining the “Positively accepted” profile, while all the categories negatively connoted were over-represented in the second cluster defining the “Negatively accepted” profile. The first cluster gathered 81% of the evaluations.

**Table III:** Clusters Description Defining “Positively Accepted” and “Negatively Accepted” Profiles

Cluster 1 - “Positively accepted”			
Category significantly over-represented	Clust/Cat^a^	Cat/Clust^b^	v.test
No alteration	91	97	19.0
No food/drink	92	93	18.2
No divided dose	88	95	13.8
No restraint	85	100	13.1
Short time	94	51	10.4
Neutral reaction	87	83	9.7
Fully taken	82	98	5.9
Positive reaction	90	12	2.8
Cluster 2 - “Negatively accepted”			
Category significantly over-represented	Clust/Cat	Cat/Clust	v.test
Use alteration	84	59	19.0
Use food/drink	69	68	18.2
Use divided dose	67	46	13.8
Negative reaction	67	45	13.6
Use restraint	100	24	13.1
Long time	40	45	8.4
Partly taken	57	10	5.1
Not taken	100	2	3.2
Medium time	24	41	2.5

^a^Percentage of all evaluations with the category belonging to the cluster

^b^Percentage of all evaluations belonging to the cluster with the category

### Model Reliability

The sampling distribution of the inertia explained by the three-dimensional map under the null hypothesis was based on 10,000 permutation rounds. The inertia value ranged from 31.9 to 34.8, with a mean of 33.3 (SD 0.4). There was no rearrangement where the statistic was superior or equal to the observed inertia value (47.2%). The null hypothesis could be clearly disproved and consequently the combinations of categories obtained from each completed evaluation questionnaire were not due to chance. Higher inertia value represents greater data set structure, therefore the reliability of the model.

The RV coefficient was significantly higher than 0 (no correlation) in all the 10,000 resampling rounds. The under-used category “Not taken” was not represented through 1.8% of the simulated data sets. In such exceptional cases the mean value of the RV coefficient evaluating the likeness between the coordinates on the maps of the remaining categories, was 0.951 [0.948; 0.955]. For other rounds, the RV coefficient mean value was 0.948 [0.947; 0.949]. The averaged maximum Jaccard coefficient were 0.966 [0.965;0.967] for the first cluster defining the ‘Positively accepted’ profile and 0.873 [0.870;0.876] for the second cluster defining the ‘Negatively accepted’ profile.

### Acceptability Scoring

**Fig. 2:**
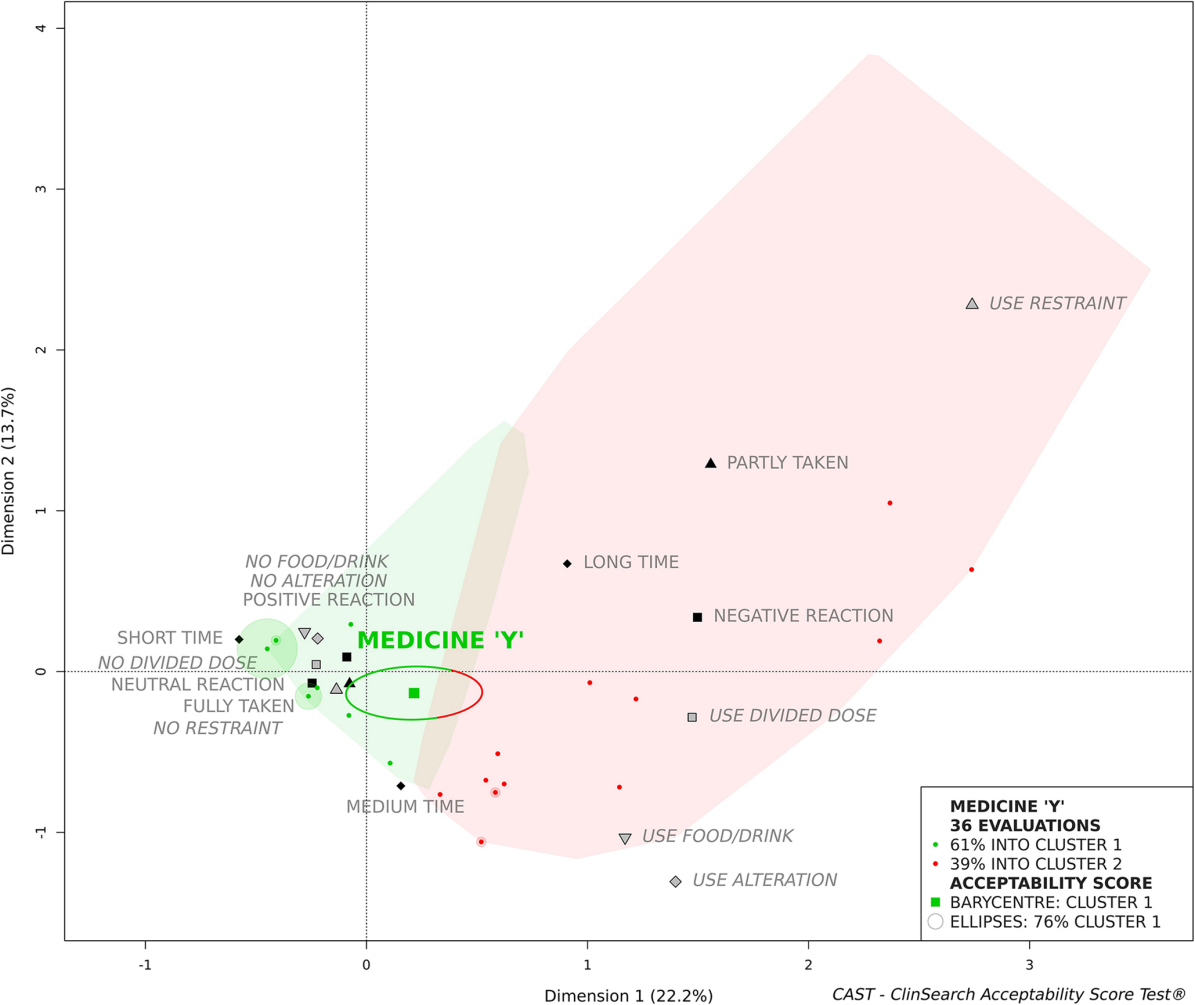
Medicine “Y” acceptability score (zoom on the acceptability map)

We focused on a medicinal product anonymously labelled medicine “Y” to illustrate acceptability scoring. Figure 2 presents the acceptability score of this psycholeptic drug as it was assessed in 36 patients. The green and the red zones on the acceptability map defined the “Positively accepted” and the “Negatively accepted” profiles, respectively. Even if the barycentre of these evaluations was assigned to the “Positively accepted” profile, this tablet cannot be assigned to this profile as a significant part of the confidence ellipses belong to the second cluster.

Although the ideal combination was the most frequently observed, accounting for 31% of the evaluations, almost two-thirds of the 19 combinations used gathered into the second cluster as “Negatively accepted”, accounting for 39% of the evaluations. This heterogeneity of evaluations brought us to explore acceptability differences in subpopulations of patients.

### Factors Affecting Acceptability

**Fig. 3:**
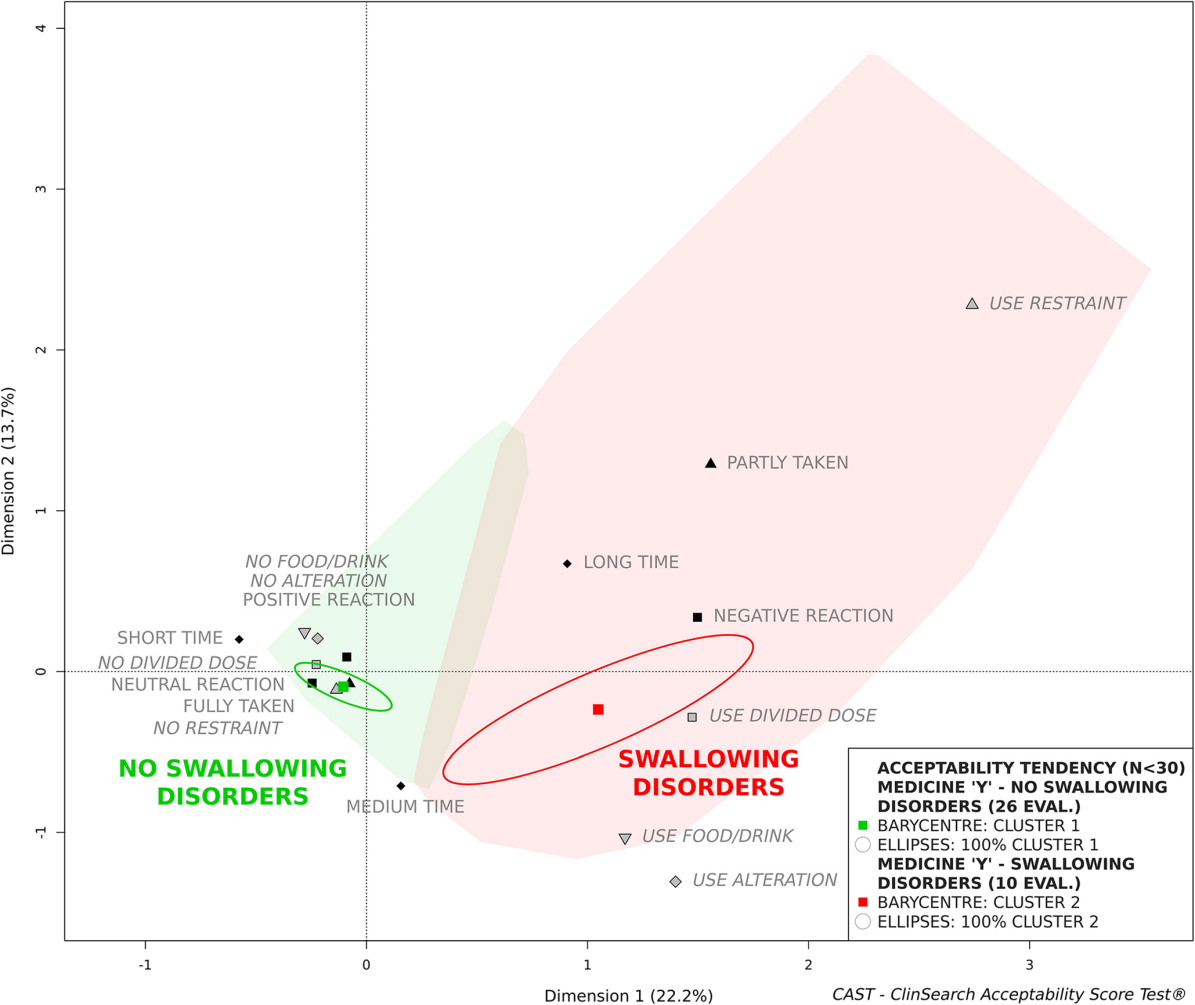
Influence of swallowing disorder on acceptability score of medicine “Y” (zoom on the acceptability map)

As age-related swallowing alteration is a major issue affecting SODF administration, we studied the influence of swallowing disorders on the medicine “Y” acceptability to illustrate explorations of factors affecting acceptability. Due to the insufficient number of patients in each sub-population (*n* < 30) we cannot draw any conclusions, nonetheless, Fig. 3 illustrates that the acceptability tended to be negatively affected by swallowing alteration. Indeed, the medicine “Y” tended to be accepted in those older patients without a swallowing disorder, while not in those with a swallowing disorder. The larger confidence ellipses observed here reflect the heterogeneity of the small group of evaluations placed on the acceptability map.

Differences in the acceptability scores reflect the significant differences observed for all the constituting variables using Fisher’s exact test for count data, with the exception of manipulation and administration time and divided dose (Table IV). Negatively connotated categories were found to be used in patients with swallowing disorders more often than their counterparts with no such disorder. As an example, there were alterations of the dosage form for 19% of the patients without swallowing disorders (60% tablet crushed, 20% tablet halved, and 20% tablet chewed), against 80% for the patients with swallowing disorders (100% tablet crushed).

**Table IV:** Measures Related to Medicine “Y” in Patients with and without Swallowing Disorder

	No swallowing disorder (*n* = 26)	Swallowing disorder (n = 10)	Fisher’s Exact Test
Result of the intake			
Fully taken	26 (100) ^*a*^	7 (70)	0.02
Partly taken	0 (0)	3 (30)	
Not taken	0 (0)	0 (0)	
Patient’s reaction			
Positive reaction	4 (15)	0 (0)	0.04
Neutral reaction	20 (77)	6 (60)	
Negative reaction	2 (8)	4 (40)	
Manip. and admin. Time			
Short time	13 (50)	2 (20)	0.18
Medium time	10 (38)	5 (50)	
Long time	3 (12)	3 (30)	
Divided dose			
Use	4 (15)	5 (50)	0.08
No	22 (85)	5 (50)	
Alteration			
Use	5 (19)	8 (80)	0.001
No	21 (81)	2 (20)	
Food/drink			
Use	3 (12)	6 (60)	0.01
No	23 (88)	4 (40)	
Restraint			
Use	0 (0)	3 (30)	0.02
No	26 (100)	7 (70)	

^a^number and percentage: n(%)

Other than swallowing alteration, there were no significant differences between these two subpopulations reinforcing the impact of the studied patient characteristic on acceptability.

## Discussion

The reference framework appears to be a valid and relevant tool to score the multi-dimensional concept of acceptability in the elderly in institutional care. The acceptability reference framework is composed of an acceptability map providing a unique Euclidian space where elements are positioned, and two acceptability profiles (“Positively accepted” and “Negatively accepted”) giving meaning to every position within the coordinate system. Acceptability could be scored using at least 30 evaluations from the standardised questionnaire. These results obtained using the method developed in the paediatric population (19,20) validate the appropriateness of this multivariate approach to address evaluation of acceptability defined as a multi-faceted concept. Furthermore, the reference framework efficacy to demonstrate the impact of both user and medicine characteristics on acceptability has been highlighted.

The acceptability reference frameworks in paediatric and older populations shared a great deal of similarities. The categories positively connoted and the related evaluations were found adjacent to one another on both three-dimensional maps, clearly separated from the categories negatively connoted and the related evaluations. Two coherent clusters defining meaningful acceptability profiles, as positive or negative, emerged in both populations. A partition with two clusters provides an effective cut-off and consequently a relevant judgement criterion. In both populations the first cluster gathered more than two-thirds of the evaluations.

Permutation testing validated robustness and significance of the relationships among categories highlighted by the acceptability map, and bootstrapping demonstrated a high stability of the map and a good recovery of the two clusters regardless of variations in the set of evaluations. These results establish the reliability of the acceptability reference framework in the older population, as in the paediatric population (20).

Although a larger set of evaluations was collected in the older than in the paediatric populations, fewer combinations of categories were used (106 vs 138). In this study we did not include medicine uses at home. Thus, some user behaviours specifically related to this setting could be missing from our current data. As such, most of the patients had no need to handle the primary and secondary medicine containers as the required doses were dispensed by healthcare professionals. In the paediatric population, the following categories were observed more often in hospital (*n* = 214) than at home (*n* = 466): “Fully taken” (93 vs 77%), “Neutral reaction” (42 vs 33%), “Short time” (75 vs 41%), “No food/drink” (91 vs 72%), “No reward” (98 vs 84%), and “No restraint” (84 vs 76%). The differences in the observational measures between these two subpopulations reflect these differences between medicine uses at home and in hospital. Indeed, the required dose was fully taken significantly more often in the older patients compared with the paediatric group (96 vs 82%). Likewise, the reaction was neutral (76 vs 36%), the preparation and administration time lower or equal to one minute (78 vs 52%) and the absence of restraint (95 vs 78%) were all more favourably scored in the older population. These differences may have highlighted the healthcare professionals’ operational capabilities in the preparation and administration of medicines. Nurses reported that they returned at a later time, sometimes without the observer, when faced with difficult patients refusing their treatments during the medicines delivery round: caregivers in the hospital setting may have thus lowered use of the category “Not taken”. As a consequence, the positively connoted categories were closer to the zero of this map, while the category “Not taken” is farther from zero than it had been placed onto the paediatric acceptability map.

In the paediatric population the required dose was divided more often than in this study (43 vs 14%). This emphasised the need for flexible dosage formats, and suitable measuring devices for children, all the more so as the required dose is age-related. There was a very low frequency of medicine alteration observed in the paediatric study, therefore the variable has not yet been included in the multivariate analysis. This could be due to a lower proportion of SODF evaluations when compared to the older population (7% against 63%), as tablets and capsules were more frequently altered for the elderly (74% of “Use alteration”). Similarly, the larger proportion of SODF in the older population could explain a shorter manipulation and administration time observed (78% inferior or equal to 1 min) compared with the paediatric population (52%). Indeed, in the older people the SODF manipulation and administration time was inferior or equal to 1 min for 89% of evaluations.

Age-related swallowing alteration is a major issue affecting the overall ability and willingness of the patient to use some medicines as intended. Crushing tablets and opening capsules are commonly used to achieve administration in patients with a swallowing disorder (4,6). That was verified by the negative impact of swallowing disorders on the acceptability score of medicine “Y”. Indeed, this tablet was crushed for 80% of the patients with a swallowing disorder. Alteration was related to a substantial increase of a required dose not fully taken, a longer preparation and administration time, a negative reaction of the patient and the use of methods to achieve administration. Even if additional data are still needed, another psycholeptic tablet mostly taken by patients without swallowing disorders (9/10) tended to be “Positively accepted” corroborating these results. Furthermore, psycholeptic oral solutions, when used in patients without swallowing disorders (12/14), tended to be “Negatively accepted”. This suggests that, even if swallowability remains crucial in oral medicine acceptability, especially in older patients with swallowing alteration, other aspects of oral medicine such as palatability drive this multi-dimensional concept. Further investigations have been carried out to identify the formulations best accepted by patients with swallowing disorders and to highlight the critical aspect of palatability in the acceptability of oral liquid preparations.

This study underlines the need to consider the specific features of each targeted users to prescribe or develop a medicine with the best adapted characteristics to reach an optimal acceptability. The acceptability reference framework could provide us with relevant knowledge on such factors positively affecting this multi-dimensionnal concept, and so appears to be a relevant decision support tool for medicinal product designers and healthcare profesionnals.

## Conclusion

This article presents a novel tool assessing the multi-dimensional concept of medicines acceptability in the elderly in institutional care. Based on a large set of medicine use evaluations combining several objective measures, an acceptability reference framework has been developed. Medicines, as well as user or product characteristics, may be positioned on a three-dimensional acceptability map and assigned to an acceptability profile: “Positively accepted” or “Negatively accepted”. The reliability and relevancy of this acceptability reference framework validate the multivariate approach transposed from the paediatric population. Due to the wide range of medicines available on the global market, and the large variety of users, additional data are being collected to continuously improve our knowledge on this complex phenomenon through the investigation of further settings, pathologies and formulations. This decision support tool facilitates the identification of those medicine features that best fit user characteristics in order to ensure the choice of appropriate formulations for adequate patient acceptability.

## Abbreviations

API: Active Pharmaceutical Ingredient
ATC: Anatomical Therapeutic Chemical classification system
EMA: European Medicine Agency
FDA: Food and Drug Administration
IADL: Lawton’s Instrumental Activities of Daily Living
ICH: International Council for Harmonization of Technical Requirements for Pharmaceuticals for Human Use
MCA: Multiple Correspondence Analysis
MD: Missing Data
MMSE: Mini Mental State Examination
SmPC: Summary of Product Characteristics
SODF: Solid Oral Dosage Form

## References

[1] Stegemann S, Gosch M, Breitkreutz J (2012). Swallowing dysfunction and dysphagia is an unrecognized challenge for oral drug therapy. Int J Pharm.

[2] Lindgren S, Janzon L (1991). Prevalence of swallowing complaints and clinical findings among 50-79-year-old men and women in an urban population. Dysphagia.

[3] Nilsson H, Ekberg O, Olsson R, Hindfelt B (1996). Quantitative aspects of swallowing in an elderly nondysphagic population. Dysphagia.

[4] Kelly J, Wright D (2009). Administering medication to adult patients with dysphagia. Nurs Stand.

[5] Caussin M, Mourier W, Philippe S, Capet C, Adam M, Reynero N, Jouini C, Colombier AS, Kadri K, Landrin I, Gréboval E, Rémy E, Marc F, Touflet M, Wirotius F, Delabre N, le Hiress C, Rorteau V, Vimard M, Dufour M, Tharasse C, Dieu B, Varin R, Doucet J (2012). L’écrasement des médicaments en gériatrie: une pratique «artisanale» avec de fréquentes erreurs qui nécessitait des recommandations. La Revue de médecine interne.

[6] Schiele JT, Penner H, Schneider H, Quinzler R, Reich G, Wezler N, Micol W, Oster P, Haefeli WE (2015). Swallowing tablets and capsules increases the risk of penetration and aspiration in patients with stroke-induced dysphagia. Dysphagia.

[7] Standing JF, Khaki ZF, Wong IC (2005). Poor formulation information in published pediatric drug trials. Pediatrics.

[8] Simojoki K, Lillsunde P, Lintzeris N, Alho H (2010). Bioavailability of buprenorphine from crushed and whole buprenorphine (subutex) tablets. Eur Addict Res.

[9] Lippert C, Gbenado S, Qiu C, Lavin B, Kovacs SJ (2005). The bioequivalence of telithromycin administered orally as crushed tablets versus tablets swallowed whole. J Clin Pharmacol.

[10] Carrier M-N, Garinot O, Stability VC (2004). Compatibility of tegaserod from crushed tablets mixed in beverages and foods. Am J Health Syst Pharm.

[11] Verrue C, Mehuys E, Boussery K, Remon JP, Petrovic M (2011). Tablet-splitting: a common yet not so innocent practice. J Adv Nurs.

[12] Johnson DA, Roach AC, Carlsson AS, Karlsson AA, Behr DE (2003). Stability of esomeprazole capsule contents after in vitro suspension in common soft foods and beverages. Pharmacotherapy: The Journal of Human Pharmacology and Drug Therapy.

[13] European Medicine Agency. Guideline on pharmaceutical development of medicines for paediatric use. 2013.

[14] European Medicine Agency. Draft - reflection paper on the pharmaceutical development of medicines for use in the older. Population. 2017;

[15] Food & Drug administration. Food and drug administration safety and innovation act (FDASIA). 2012.

[16] International Council for Harmonisation. ICH E11(R1) guideline on clinical investigation of medicinal products in the pediatric. Population. 2016;

[17] Kozarewicz P (2014). Regulatory perspectives on acceptability testing of dosage forms in children. Int J Pharm.

[18] Drumond N, van Riet-Nales DA, Karapinar-Carkit F, Stegemann S. Patients' appropriateness, acceptability, usability and preferences for pharmaceutical preparations: results from a literature review on clinical evidence. Int J Pharm 2017. Epub 2017/02/24, 521, 294, 305. 10.1016/j.ijpharm.2017.02.029 28229945

[19] Ruiz F, Vallet T, Pense-Lheritier AM, Aoussat A (2017). Standardized method to assess medicines' acceptability: focus on paediatric population. J Pharm Pharmacol.

[20] Vallet T, Ruiz F, Lavarde M, Pense-Lheritier AM, Aoussat A (2018). Standardized evaluation of medicine acceptability in paediatric population: reliability of a model. J Pharm Pharmacol.

[21] Food & Drug administration. Guidance for industry: patient-reported outcome measures: use in medical product development to support labeling claims.. 2009. 10.1186/1477-7525-4-79 PMC1629006 17034633

[22] Matza LS, Patrick DL, Riley AW, Alexander JJ, Rajmil L, Pleil AM, Bullinger M (2013). Pediatric patient-reported outcome instruments for research to support medical product labeling: report of the ISPOR PRO good research practices for the assessment of children and adolescents task force. Value Health.

[23] Lawton MP, Brody EM (1970). Assessment of older people: self-maintaining and instrumental activities of daily living. Nurs Res.

[24] Fried LP, Tangen CM, Walston J, Newman AB, Hirsch C, Gottdiener J, Seeman T, Tracy R, Kop WJ, Burke G, McBurnie MA (2001). Frailty in older adults: evidence for a phenotype. J Gerontol Ser A Biol Med Sci.

[25] Derouesné C, Poitreneau J, Hugonot L, Kalafat M, Dubois B, Laurent B (1999). Le Mini-Mental State Examination (MMSE): un outil pratique pour l'évaluation de l'état til pratique pour l'évaluation cognitif des patients par le clinicien version française consensuelle. Presse Med.

[26] Le S, Josse J, FactoMineR HF (2008). An R package for multivariate analysis. J Stat Softw.

[27] Josse J, missMDA HF (2016). A package for handling missing values in multivariate data analysis. J Stat Softw.

[28] Good P (2000). Permutation tests springer New York.

[29] Efron B, Tibshirani RJ (1994). An introduction to the bootstrap: CRC press.

[30] Robert P, Escoufier YA (1976). Unifying tool for linear multivariate statistical methods: the RV- coefficient. J R Stat Soc: Ser C: Appl Stat.

[31] Hennig C (2007). Cluster-wise assessment of cluster stability. Comput Stat Data Anal..

[32] Josse J, Pages J, Husson F (2008). Testing the significance of the RV coefficient. Comput Stat Data Anal.

